# Probing oculomotor inhibition with the minimally delayed oculomotor response task

**DOI:** 10.1007/s00221-018-5345-9

**Published:** 2018-07-30

**Authors:** Paul C. Knox, Emma Heming De-Allie, Felicity D. A. Wolohan

**Affiliations:** 1 Eye and Vision Science, Institute of Ageing and Chronic Disease, University of Liverpool, William Henry Duncan Building, 6 West Derby Street, Liverpool, L7 8TX UK; 2 Department of Psychology, Edge Hill University, Ormskirk, UK

**Keywords:** Saccade, Antisaccade, Inhibition, Latency, Gap, Overlap

## Abstract

The ability to not execute (i.e. to inhibit) actions is important for behavioural flexibility and frees us from being slaves to our immediate sensory environment. The antisaccade task is one of several used to investigate behavioural inhibitory control. However, antisaccades involve a number of important processes besides inhibition such as attention and working memory. In the minimally delayed oculomotor response (MDOR) task, participants are presented with a simple target step, but instructed to saccade not to the target when it appears (a prosaccade response), but when it disappears (i.e. on target offset). Varying the target display duration prevents offset timing being predictable from the time of target onset, and saccades prior to the offset are counted as errors. Antisaccade error rate and latency are modified by alterations in fixation conditions produced by inserting a gap between fixation target offset and stimulus onset (the gap paradigm; error rate increases, latency decreases) or by leaving the fixation target on when the target appears (overlap paradigm; error rate decreases, latency increases). We investigated the effect of gaps and overlaps on performance in the MDOR task. In Experiment 1 we confirmed that, compared to a control condition in which participants responded to target onsets, in the MDOR task saccade latency was considerably increased (increases of 122–272 ms depending on target display duration and experimental condition). However, there was no difference in error rate or saccade latency between gap and synchronous (fixation target offset followed immediately by saccade target onset) conditions. In Experiment 2, in a different group of participants, we compared overlap and synchronous conditions and again found no statistically significant differences in error rate and saccade latency. The timing distribution of errors suggested that most were responses to target onsets, which we take to be evidence of inhibition failure. We conclude that the MDOR task evokes behaviour that is consistent across different groups of participants. Because it is free of the non-inhibitory processes operative in the antisaccade task, it provides a useful means of investigating behavioural inhibition.

## Introduction

Behavioural inhibition is the ability to stop various categories of actions (Aron 2007). It is a key component of executive function and is vital for behavioural flexibility (Friedman and Miyake 2017; Miyake et al. 2000). A wide range of tasks have been used to investigate behavioural inhibition including manual response tasks such as the go/no go (Donders 1969) and stop signal (Verbruggen and Logan 2008) tasks, and a particular oculomotor task, the antisaccade task (Hallett 1978; Hutton and Ettinger 2006; Munoz and Everling 2004).

Saccades are closely linked with, and reflective of, various aspects of cognition and thus provide a means of investigating key components of executive function such as attention, working memory and (of particular significance for the current study) behavioural inhibition. They are easily elicited and recorded and can be described in terms of parameters such as latency, amplitude, peak velocity and duration and in the case of antisaccades, the error rate (Leigh and Kennard 2004). Their underlying neurophysiology is well understood, having been established in non-human primates by means of single unit recording (including in awake, behaving conditions) and functional imaging in humans (Berman et al. 1999; Brown et al. 2008; McDowell et al. 2008; Munoz and Everling 2004).

In the antisaccade task, participants are usually presented with a stimulus in which a target appears to the left or right of fixation, but are instructed to look to the mirror image position of that target position. The directional error rate (the proportion of trials in which participants look at the target rather than to the instructed position) is taken to provide a measure of inhibition, with high error rates implying poor inhibitory control. While successful antisaccade performance certainly requires the inhibition of the normal, reflexive, saccade response to the target position, it also requires the computation and execution of a voluntary saccade to the instructed position. Competition between reflexive pro- and voluntary antisaccade processes (Reuter et al. 2005), and the involvement of attention (Gaspelin and Luck 2018) and working memory (Crawford et al. 2011) in this task mean that interpreting poor antisaccade performance as only indicating an inhibitory control deficit is problematic.

We have, therefore, used a simple variant of a prosaccade task, the “minimally delayed oculomotor response” (MDOR) task, to investigate oculomotor inhibition. The stimulus is a target step with randomised direction and timing, and a variable target display duration. Participants are instructed to execute their saccade to the target, but critically not when it appears (a reflexive prosaccade response), but when the target is extinguished. Thus, as in the antisaccade task it is necessary to inhibit the normal reflexive response to a target onset. Unlike the antisaccade task, no vector inversion is required (the eventual saccade is to the actual target position). And unlike other types of delayed response or memory-guided tasks, the working memory requirement is minimal (the target is present throughout the period of central fixation). No position other than the actual target position needs be attended to, to which we assume attention is drawn by the saccade target onset. Performance is measured using the error rate (the proportion of trials in which participants execute a target-directed saccade prior to target offset) and the latency of correct responses.

We previously used the MDOR task to investigate oculomotor inhibition in “express saccade makers” (ESMs), participants who execute very high proportions of express saccades even in conditions designed to discourage this type of response (Amatya et al. 2011; Cavegn and Biscaldi 1996). These participants also have increased antisaccade directional error rates (Biscaldi et al. 1996; Knox et al. 2012). However, when tested on the MDOR task, their performance was identical to a non-ESM control group, and across participants we found no correlation between the antisaccade and MDOR error rates (Wolohan and Knox 2014). In these experiments, we used a synchronous version of the MDOR task in which the saccade target appeared when the fixation target was extinguished.

In the current study, our objective was to investigate the MDOR task further. Performance on the antisaccade task is modulated by fixation state. In particular, the error rate is increased if a gap is introduced prior to target appearance (Fischer and Weber 1997). Here we investigated whether this is also true of the MDOR task. In Experiment 1, we first confirmed the basic pattern of results for the MDOR task as reported previously (Wolohan and Knox 2014) by comparing MDOR and control tasks, and then compared gap and synchronous versions of the task. In Experiment 2, with a new group of participants, we compared synchronous and overlap versions of the task. Finally, as there are little published data on the MDOR task, we compared the previously published synchronous data from Wolohan and Knox (2014) with data from Experiments 1 and 2.

## Methods

### Ethics and participants

Healthy, adult participants, with normal or corrected to normal vision were recruited from the University community, under ethical approval from the University of Liverpool Research Ethics committee. All gave informed, written consent after the experiment was explained and they had an opportunity to ask questions. A total of 35 participants were recruited in the current study. Twenty naïve participants (mean age 22 years, range 19–40 years; 12 female) took part in Experiment 1 (comparison of gap and synchronous MDOR tasks, with gap and synchronous control data). Fifteen different participants (mean age 29 years, range 19–50 years; six female) took part in Experiment 2 (comparison of overlap and synchronous tasks); seven of these participants were naïve to oculomotor testing, while eight had participated in our previous MDOR study (Wolohan and Knox 2014) in which they had only been exposed to synchronous tasks.

### Apparatus and stimuli

To allow comparisons to be made between the present experiments and those of Wolohan and Knox (2014), we used the same apparatus and stimuli as used in that earlier study for MDOR experiments. Briefly, stimuli were presented on a 21″ monitor (1024 × 768 spatial resolution, 100 Hz temporal resolution) driven by a VSG2/5 card (Cambridge Research Systems, Rochester, UK), positioned on the fronto-parallel plane 57 cm from the participant’s eye. Horizontal eye position of the left eye was recorded using a Skalar Iris IR Eye Tracker, with the eye tracker output digitised with 16-bit precision using a CED Power 1401 (Cambridge Electronic Design, Cambridge, UK) interface. Oculomotor data were stored for off-line, trial-by-trial analysis using custom software.

**Fig. 1:**
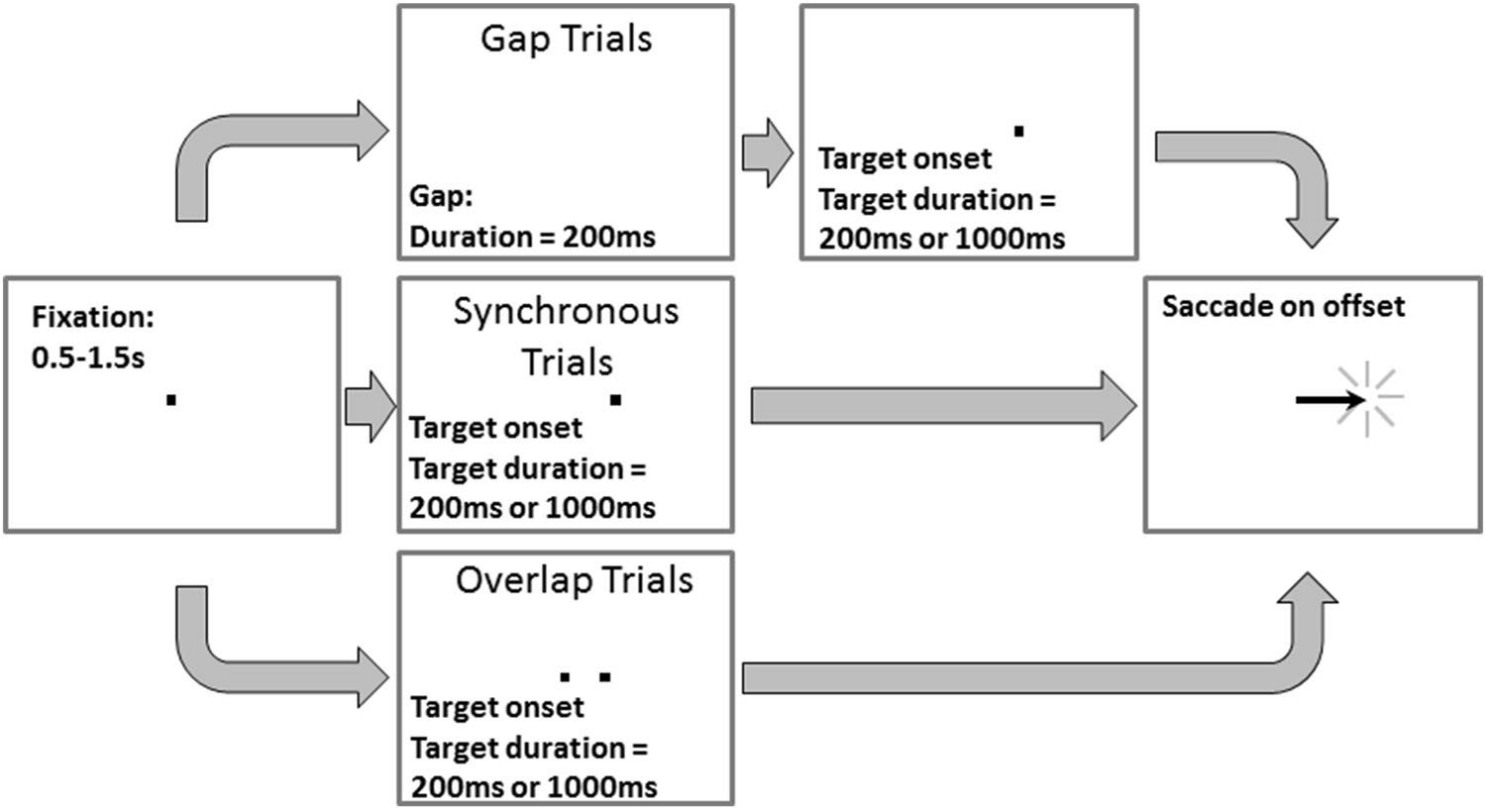
Illustration of MDOR trial types. Note that different types were presented in different blocks

Three types of MDOR trial were run in the current study (Fig. 1). In synchronous tasks (as used by Wolohan and Knox 2014), a central fixation target (0.2° black square) was presented on a light background for a randomised period of 0.5–1.5 s. Immediately when it was extinguished, the saccade target (a 0.2° black square) appeared 5° to the left or to the right of fixation (randomised and with equal frequency) and was displayed for either 200 or 1000 ms (randomised from trial to trial). These stimulus conditions are identical to what are often called step trials in other studies. Participants were instructed to maintain fixation in the centre of the display until the saccade target disappeared, when they were to execute a saccade to the target’s location (i.e. saccade on target offset), pause, and return their gaze to the central point in preparation for the next trial. They were explicitly instructed not to saccade to the onset of the target. Gap MDOR tasks were identical except that between fixation target extinction and saccade target appearance, there was a blank period (i.e. the gap) of 200 ms (Fig. 1). In contrast, in overlap MDOR tasks the fixation target was not extinguished, and remained visible for the duration of the trial. Different trial types were presented in different blocks. Experiment 1 consisted of synchronous and gap MDOR blocks and control blocks. Control blocks were composed of synchronous or gap trials, but participants were instructed to saccade on target onset (i.e. execute the normal prosaccade response to target appearance). In Experiment 2, synchronous and overlap blocks were run.

### Procedures

Participants were carefully positioned by adjusting table height, a chin rest and cheek pads. In Experiment 1 they were then exposed to runs of gap MDOR and control, and synchronous MDOR and control trials (120 trials per run; run order counterbalanced across participants). In Experiment 2 participants completed runs of 2 × 120 overlap MDOR and 2 × 120 synchronous MDOR trials. The quality of performance was carefully monitored to ensure that it was maintained, with verbal feedback given as necessary. After either a single block, or no more than two blocks, a 32-trial calibration procedure was performed.

In calibration trials, after a randomised fixation period (0.5–1.5 s), the fixation target was extinguished and a saccade target was presented to the left or to the right with an eccentricity of 5° or 10° (randomised and with equal frequency) for 1 s. Participants were instructed to fixate the central point and saccade to the target as soon as it appeared, fixating it until it was extinguished, at which point they could blink and return to the centre, ready for the next trial.

### Analysis

Data were analysed using an interactive programme which displayed the eye position data and the time at which the “go” signal appeared. This was the target offset in the MDOR tasks and the target onset in control tasks. The calibration data were used to transform the data from arbitrary system units into units of degrees of eye rotation. Trials with blinks or unstable fixation prior to target appearance were removed from the analysis. In Experiment 1, the mean yield of trials for analysis was 87 and 86% for synchronous MDOR and control runs, respectively, and 94 and 92% for gap MDOR and control runs. In Experiment 2, the mean yield was 87% for synchronous and 93% for overlap MDOR runs. For each primary saccade, a cursor was placed at the offset (MDOR task) or onset (Control task) of the target and then at the beginning of the saccade (judged by eye) to calculate latency. For amplitude, the cursor was placed at the initial eye position and then at the end of the saccade, and amplitude was calculated as the difference between the first and second position measurements.

Data were collated in MS Excel. Error responses were first identified, removed and collated separately from correct trials, and the error rate calculated. In MDOR trials, any target-directed saccade with an amplitude greater than 1° that occurred from 80 ms after target onset to 80 ms after target offset was counted as an error. Any target-directed saccade occurring from 80 to 600 ms after target offset was counted as a correct response. For each participant, median saccade latency was calculated along with error rate. Error and latency data for the two target display durations (200 and 1000 ms) were kept separate. Statistical analysis (details in “Results”) was conducted with SPSS v22.

## Results

### Experiment 1: Comparison of gap and synchronous MDOR tasks

**Fig. 2:**
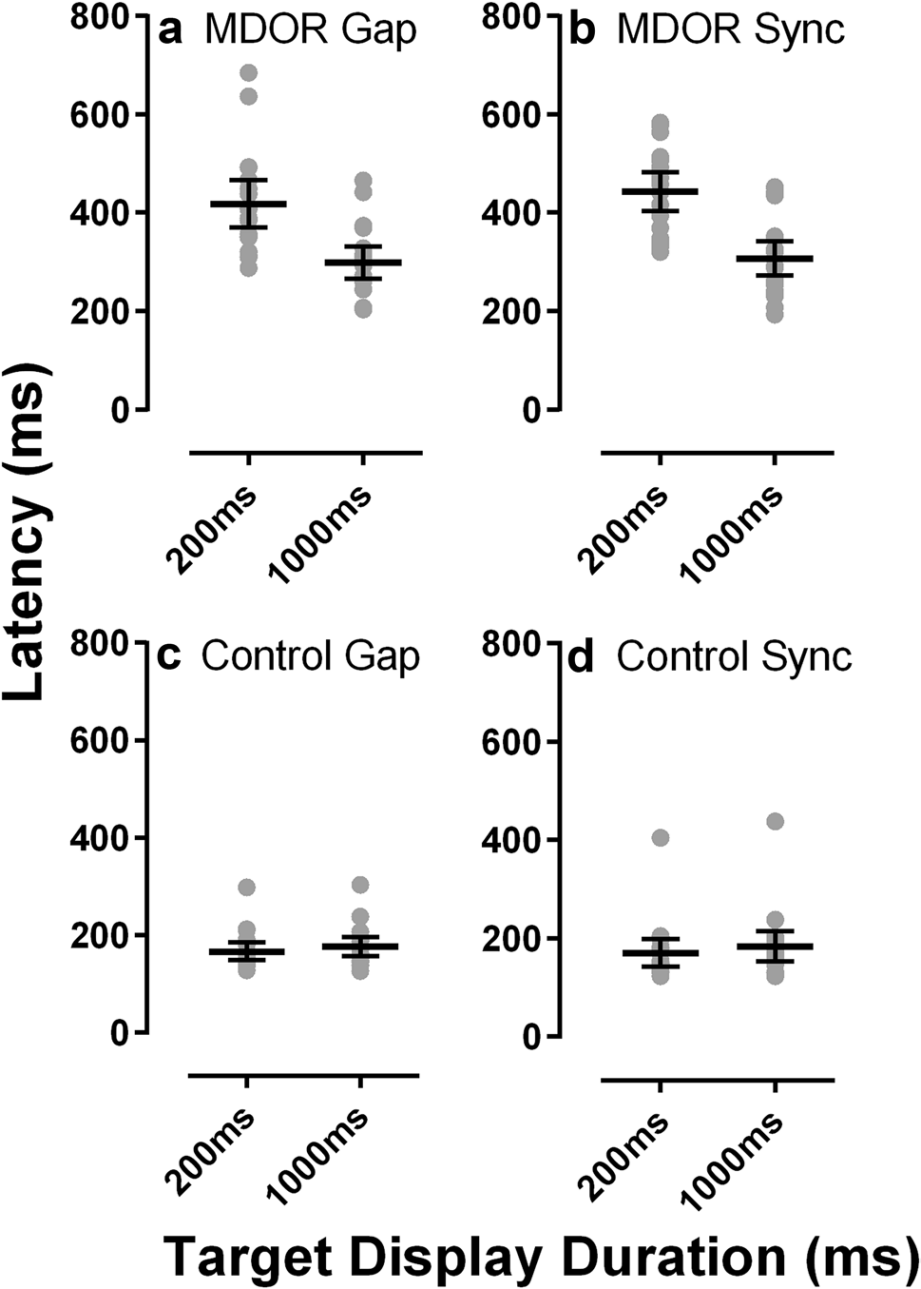
Comparison of performance between gap and synchronous MDOR tasks (**a, b**) and gap and synchronous control tasks (**c, d**). Points show individual median latency for each participant; horizontal lines indicate intersubject mean ± 95% CI

We first confirmed that the basic pattern of performance observed for the MDOR task was similar to that reported previously. As illustrated in Fig. 2, compared to the control condition (Fig. 2c, d) in which participants executed a simple prosaccade response to target onsets, latency for saccades executed to the offset of targets was considerably increased in both gap (Fig. 2a) and synchronous tasks (Fig. 2b). We also observed a modulation in latency dependant on the target display duration in MDOR tasks. Thus for MDOR synchronous tasks, mean intersubject latency (± SD) was 442 ± 84 and 307 ± 74 ms for target display durations of 200 and 1000 ms compared to 171 ± 60 and 183 ± 65 ms in the relevant control conditions. Table 1 shows the mean difference in latency between gap and synchronous MDOR tasks and the relevant control condition across participants. We analysed the difference between the MDOR task results and the control conditions using separate repeated measures ANOVA’s with identical design, treating target display duration (200 vs 1000 ms) and task type (MDOR vs control) as within subjects factors. As might be expected from Fig. 2, both factors had a statistically significant effect on saccade latency (gap: display duration *F*_1,19_ = 79, *p* < 0.001; type *F*_1,19_ = 105, *p* < 0.001; synchronous: display duration *F*_1,19_ = 27, *p* < 0.001; type *F*_1,19_ = 192, *p* < 0.001). While for both gap and synchronous data there were significant interactions between display duration and task type (gap: *F*_1,19_ = 81, *p* < 0.001; synchronous: *F*_1,19_ = 70, *p* < 0.001), this is primarily because for both the modulation in latency observed in the main tasks is, unsurprisingly, not present in the control tasks.

**Table 1:** Mean (± SD) difference in latency between MDOR and control conditions for gap and synchronous tasks, shown for the two target display durations

Target duration (ms)	Condition	
	Gap	Synchronous
200	252 ± 105 ms	272 ± 77 ms
1000	122 ± 66 ms	135 ± 76 ms

This difference represents the increase in latency in the MDOR task compared to a simple reflexive prosaccade response to the same stimuli

For the gap data, simple main effects analysis indicated that for the MDOR task there was a significant effect of target duration (*p* < 0.001); mean latency for was greater for the 200 ms (419 ms) compared to 1000 ms (298 ms) target display duration. There was also significant (though much smaller) effect in the control task (*p* = 0.017); mean latency was longer for the 1000 ms (177 ms) compared to the 200-ms (167 ms) duration. For the synchronous data, simple main effects analysis indicated that for MDOR task there was a significant effect of target display duration (*p* < 0.001); mean latency was greater with a 200-ms display time compared to 1000 ms (442 vs 304 ms). There was no significant effect of duration for the control task (*p* = 0.89).

**Fig. 3:**
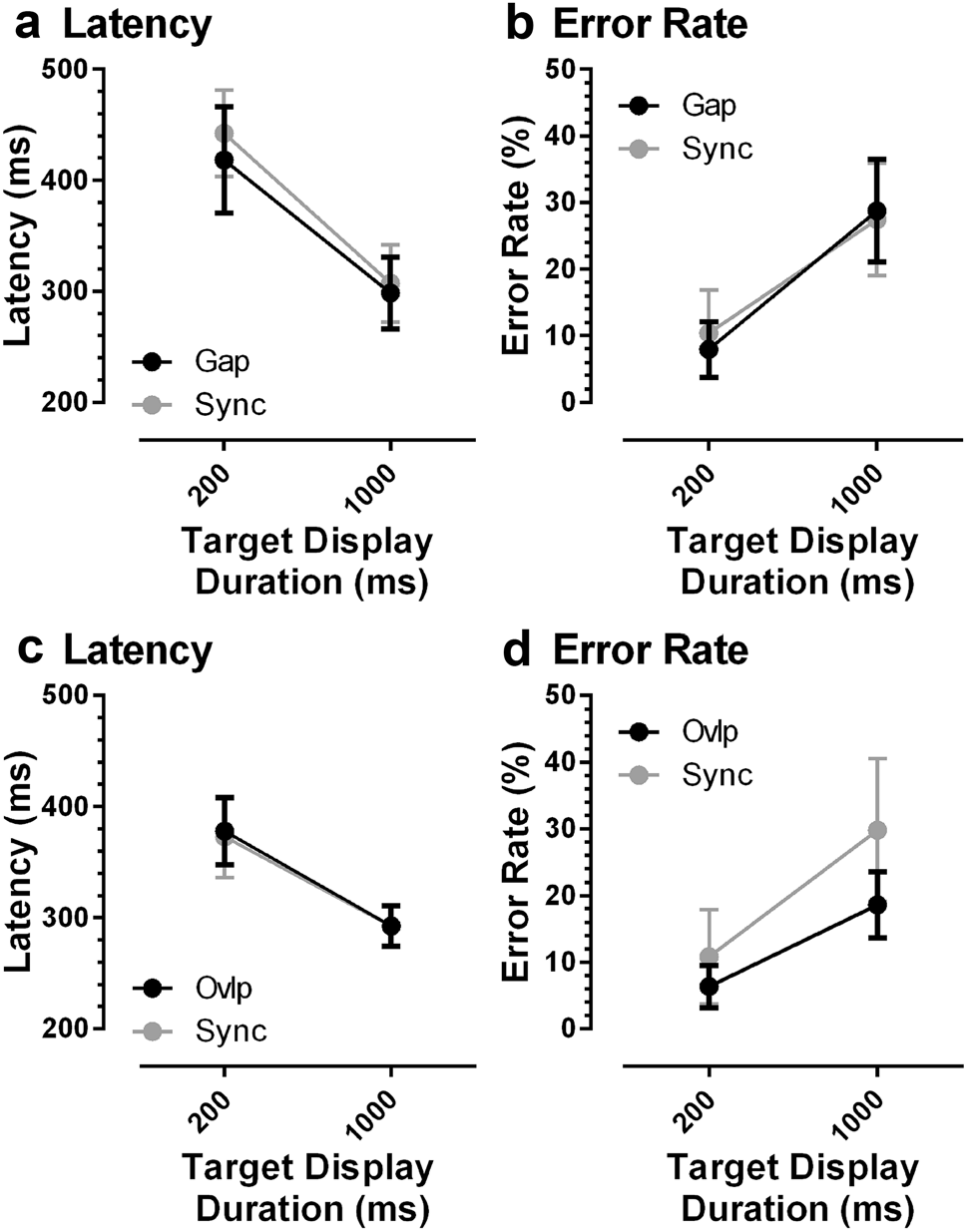
Comparison of latency (**a, c**) and error rate (**b, d**) for gap (black circle) vs synchronous (grey circle) MDOR tasks (data from “Experiment 1”) and overlap vs synchronous (**c, d**) MDOR tasks (data from “Experiment 2”). Intersubject means (± 95% CI) are shown

Our main objective was to compare gap and synchronous MDOR tasks directly (Fig. 3a, b). There was little evidence of a difference either in latency or in error rate between the two task types. When the latency data were tested with a repeated measures ANOVA of similar design to that used above, with display duration and task type as within subjects factors, display duration again returned a statistically significant result (*F*_1,19_ = 85, *p* < 0.001), task type (gap vs synchronous) did not (*F*_1,19_ = 1.6, *p* = 0.22) and there was no interaction between factors (*F*_1,19_ = 0.63, *p* = 0.44). The same result was evident for error rate: a significant effect of display duration (*F*_1,19_ = 43, *p* < 0.001), no difference between tasks (*F*_1,19_ = 0.03, *p* = 0.87) and no interaction between factors (*F*_1,19_ = 0.83, *p* = 0.37).

### Experiment 2: Comparison of overlap and synchronous MDOR tasks

In a different group of participants to those in Experiment 1, we investigated whether in overlap conditions, in which the fixation target remained visible throughout the trial, there was a difference either in latency or error rate compared to synchronous trials (Fig. 3c, d). For both latency and error rate, there was again a large modulation with display duration which in both cases was statistically significant (latency: *F*_1,14_ = 60, *p* < 0.001; error rate *F*_1,14_ = 94, *p* < 0.001). Task type did not influence latency (*F*_1,14_ = 0.08, *p* = 0.78). There did appear to be a reduction in error rate in overlap compared to gap tasks (200 ms overlap 6 ± 6% vs synchronous 11 ± 13%; 1000 ms 19 ± 9 vs 30 ± 12%). However, when tested with ANOVA there was no statistically significant difference in error rate between task types (*F*_1,14_ = 4.3, *p* = 0.06), although a significant interaction between task type and display duration was observed (*F*_1,28_ = 5.9, *p* = 0.03).

Simple main effects analysis indicated that for both overlap and synchronous task types there was a significant effect of duration (for both: *p* < 0.001). For both task types, more errors were observed with the 1000 ms compared to 200-ms target display duration (overlap:18.6 vs 6.4%; synchronous: 29.8 vs 10.8%). Analysis also indicated that for the 1000-ms condition significantly more errors were produced in the synchronous compared to overlap task (*p* < 0.05; 29.8 vs 18.6%) with no difference between task type for the 200-ms condition (*p* = 0.129).

### Comparison of synchronous data across three experiments

**Fig. 4:**
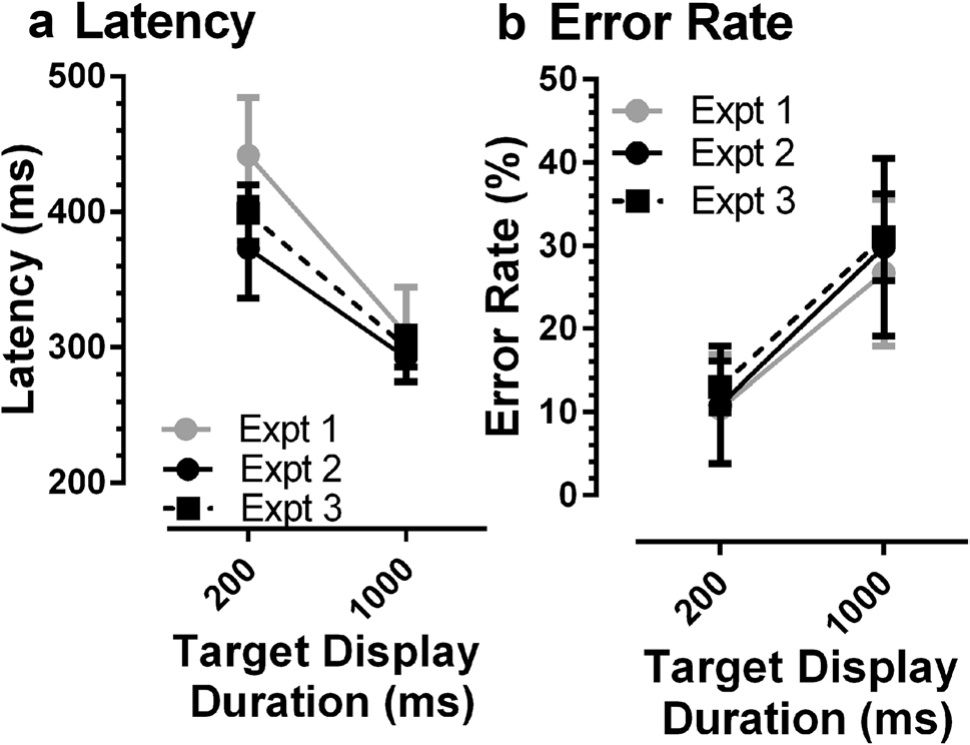
Comparison of latency (**a**) and error (**b**) data from three different groups of participants in synchronous MDOR tasks

We compared synchronous latency and error rate data from three different groups of participants: those from Experiments 1 (*N* = 20) and 2 (*N* = 15) and the participants from Wolohan & Knox (2014) who did not take part in Experiment 2 (*N* = 44; mean age 23 years, range 19–43 years; 31 female). This last group will be referred to as Experiment 3 for convenience. This analysis confirmed that across the three experiments the general pattern of results was consistent (Fig. 4). There were some differences in latency between experiments, limited primarily to the 200-ms display duration (Expt 1: 442 ± 92 ms, 95% CI 409–475 ms; Expt 2: 373 ± 66 ms, 333–413 ms; Expt 3: 399 ± 69 ms, 379–419 ms). For the 1000-ms condition latency was very similar (Expt 1: 309 ± 74 ms, 293–325 ms; Expt 2: 292 ± 32 ms, 259–325 ms; Expt 3: 300 ± 49 ms, 286–214 ms). As is clear from Fig. 4b, error rates were very similar across experiments. We analysed the latency data with a repeated measures ANOVA treating display duration as a within, and experiment as a between subjects factor. As might be expected, display duration had a statistically significant effect on latency (*F*_1,76_ = 193; *p* < 0.001) while experiment did not (*F*_2,76_ = 2.5; *p* = 0.086); there was a statistically significant interaction between the factors (*F*_2,76_ = 3.5; *p* = 0.034). The same analysis conducted on error rates demonstrated the modulation due to target display duration (*F*_1,76_ = 104; *p* < 0.001), but no statistically significant effect of experiment (*F*_2,76_ = 0.48; *p* = 0.621) and no interaction (*F*_2,76_ = 0.20; *p* = 0.82).

**Fig. 5:**
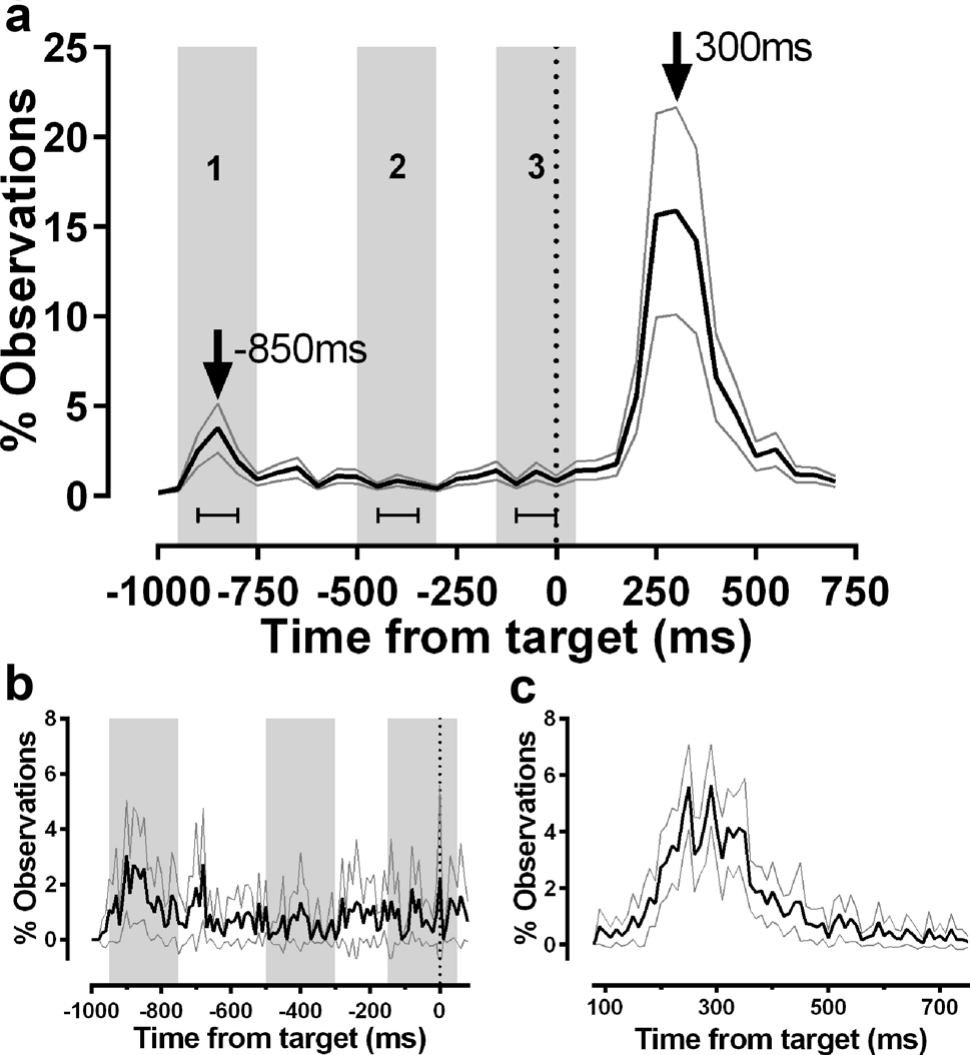
Average percentage distribution histograms of latency for the 1000-ms display duration condition from the synchronous MDOR task. Data averaged across all 20 participants from Experiment 1, plus seven naïve participants from Experiment 2. **a** Summary distribution; bin width 50 ms. For each participant, the % frequency distribution for responses from − 1200 to 1000 ms was calculated. The mean (± 95% CI) was then calculated for each bin across participants. The black central line is plotted through the each mean bin value, while grey lines show ± 95% CI. Target onset was at − 1000 ms and target offset (the go signal) at 0 ms. Arrows mark two peak bins in the distribution: − 850 ms (150 ms after target onset)—error peak; 300 ms—correct responses. The grey regions delineate three five-bin epochs; epoch 1: post-onset − 950 ms to – 750 ms; epoch 2: mid-fixation − 500 ms to − 300 ms; epoch 3: “anticipatory” − 150 ms to + 50 ms. Within each region the central three bins were used for statistical analysis. **b** Average percentage distribution plot for errors. These were recalculated for data from − 1200 to 80 ms, using a bin width of 10 ms. Other conventions as for a. Grey regions show the same timing epochs as in a, although now for a higher number of bins. **c** Average percentage distribution plot for correct responses. Data from 80 to 750 ms recalculated using a bin width of 10 ms. Other conventions as for a. These histograms are comparable with Fig. 4c, d in Wolohan and Knox (2014)

The pattern of timing of errors and correct responses was further examined by compiling average distribution histograms for the 1000-ms display duration condition for all 20 participants in Experiment 1, plus the naïve participants from Experiment 2 (Fig. 5). These histograms are comparable with Fig. 4c, d in Wolohan and Knox (2014), with no overlap in participants. The target offset (the go signal) occurred at 0 ms. To provide an overall summary of error and correct response timings, we first conducted this analysis of all responses using a bin width of 50 ms (Fig. 5a). Two peaks are evident in this distribution, one at 300 ms (composed of correct responses) and an earlier and smaller peak 850 ms prior to target offset comprised of errors. We defined three epochs within this distribution, illustrated by three numbered grey ranges in Fig. 5a. Epoch 1 consisted of five histogram bins (− 950 ms bin to − 750 ms bin), centred on the peak error bin (− 850 ms); it contained 9.49% of the responses. Epoch 2 consisted of five bins beginning at − 500 ms, in the middle of the fixation period, and contained 3.48% of the responses. Epoch 3, which was intended to capture any evidence of “anticipatory errors”, was the five bins beginning at − 150 ms, and contained 5.63% of responses. For comparison, the five bins centred on the peak capturing the correct responses at + 300 ms, contained 78.83% of responses. Epochs 1–3 were compared statistically using a repeated measured ANOVA, using data from the three centre bins of each epoch, and treating “bin” as a within subjects factor, and “epoch” (1:error vs 2:mid-interval vs 3:anticipatory) as a between subjects factor. While “bin” failed to reach statistical significance (*F*_2,83_ = 3.0; *p* = 0.051), “epoch” returned a clearly statistically significant result (*F*_2,84_ = 11.9; *p* < 0.001); post hoc testing (Tukey HSD) demonstrated that this was driven by the difference between epoch 1 and epochs 2 and 3 (*p* < 0.001 and *p* = 0.001 respectively).

An average distribution for the error responses alone was constructed, with the distribution recalculated using a bin width of 10 ms to further investigate the relative timing of errors (Fig. 5b). While necessarily noisier (represented by wide 95% CI’s), there was an early peak in this distribution at − 900 ms (i.e. 100 ms post-onset), with what appeared to be a smaller peak at − 680 ms (320 ms post-onset). There was again little evidence of a build-up of errors in the anticipatory epoch. A separate distribution for correct responses (Fig. 5c) exhibited two peaks at 250 and 290 ms.

## Discussion

Our objective in this study was to investigate the characteristics of performance on the MDOR task in normal, healthy, adult participants. In particular, we wished to establish the effect of fixation conditions (gaps and overlaps) on the latency and error rates of saccades executed in the MDOR task. The stimulus in the MDOR task is a standard, reflexive, prosaccade stimulus. However, because participants are instructed to saccade to the *offset* of the target rather than its *onset*, they must inhibit their normal response to the target onset. Varying the target display duration (in this experiment either 200 or 1000 ms) prevents participants anticipating the offset timing from the time of the onset. Resulting saccade latencies are much longer than would be expected in a reflexive paradigm.

The MDOR task is conceptually similar to an oculomotor delayed response (ODR) or memory-guided saccade (MGS) task, although with key differences (Pierrot-Deseilligny et al. 2002). Although a delayed response does not necessary imply a memory delay, in practice these two terms have generally referred to the same type of task (McDowell et al. 2008). In the MDOR task, there is no memory period in that the target is present throughout the period during which central fixation is maintained; there is nothing to be remembered. We do not know when within each trial target position is encoded. If target position is encoded at the beginning of the fixation period (after target onset but prior to target offset), then potentially this would imply a working memory load during the fixation period. But given that the target is still present, in contrast to a MGS task in which it is removed during a memory delay of typically several seconds, this is not required. Note also that in MGS tasks participants are usually explicitly instructed to remember target position. It is also possible that as it is the target offset that is the go signal, in successfully executed MDOR trials participants encode the desired position and programme the saccade in response to that offset (any response and spatial memory of the onset having been successfully inhibited), in which case the working memory load would be negligible. For the moment, we cannot distinguish between these possibilities. Using target offset as the go signal also differs from standard MGS tasks in which the offset of the fixation target is usually the go signal.

Finding comparative data in the literature, in which saccade latency in a MGS task is compared to a control task with no memory delay, is surprisingly difficult. Many reports concentrate on the spatial accuracy of memory-guided saccades rather than latency. However, Smit et al. (1987) reported latency data for four participants in which a memory delay-induced latency increases 50–100 ms compared to a simple prosaccade response. Data from one of the conditions described by Nuthmann et al. (2016) suggested a smaller latency increase of the order of 40 ms. These latency differences are smaller than those we observed in the current experiment (see Table 1).

We used a relatively simple saccade task with a single target eccentricity to ensure direct comparability with Wolohan and Knox (2014). Latency data from the control tasks (mean saccade latencies of 170 to 180 ms) suggest that the randomisation of direction, as well as the randomisation of pretarget fixation times, was sufficient to prevent the development of the type anticipatory response seen in highly predicable task contexts. More complex task types, with more spatial uncertainty (i.e. multiple eccentricities or > 2 potential target positions) would be worth investigating.

Increased latency in MDOR compared to control tasks could be due, in part, to requiring responses to offsets, which are thought to be less salient than onsets (Cole and Kuhn 2010), particularly within the context of oculomotor capture (Boot et al. 2005). However, onset/offset differences are again smaller than the effects observed with the MDOR task. For a prosaccade task, Pratt and Trottier (2005) reported this difference (the “cost” of the saccade target being an offset) to be of the order of 50–90 ms. Reuter et al. (2011) reported differences of a similar magnitude. In a more recent study, Reuter et al. (2016) reported data for synchronous and overlap prosaccade tasks in control participants for a clinical study, in which the offset/onset difference was smaller; differences of 37 and 17 ms were observed. It is unlikely that offset effects explain the magnitude of the latency increases in the MDOR task (Fig. 2; Table 1). If these were simply due to an offset effect, this would not explain the latency differences observed with different target display durations. The magnitude and pattern of latency effects are also not consistent with other mechanisms such as foreperiod effects (Findlay 1981; Van Koningsbruggen and Rafal 2009).

Larger increases in saccade latency have been reported where participants are aware that they may need to withhold (i.e. inhibit) a response. Machado and Rafal (2000) compared a standard prosaccade condition with a go/no go condition in which the nature of the saccade target indicated whether a saccade was to be made or not from trial to trial. They observed latency increases of the order of 150 ms in the go/no go condition and interpreted this as being due to inhibition of the normal reflexive response. While this paradigm is different to the MDOR task in important respects, inhibition is the key feature of both. In the MDOR task, when a target appears, participants are aware that they should not saccade to it. Therefore, around the time of target appearance we assume that levels of inhibition will be high. If the target is extinguished after a short period (200 ms in the current experiments), a saccade has to be executed, but against a high level of inhibition. It is this that leads to long latencies in the 200-ms condition, much longer than is consistent with a reflexive response. We assume the level of inhibition then begins to fall. When the target is extinguished after 1000 ms, the resultant saccade is executed against a lower level of inhibition, leading to shorter latencies compared to the 200-ms condition (although still more than 100 ms longer than control latencies). Although only two target display durations were used in the current experiments, previously we used a wider range of target display durations and found that there was a monotonic relationship between display duration and latency (Knox and Abd Razak 2010).

Inhibition of the reflexive response to a target appearance is also required in the antisaccade task, hence its widespread use as a means of measuring inhibition (Alichniewicz et al. 2013; Crawford et al. 2002, 2005; Goto et al. 2010). However, in the antisaccade task other processes such as working memory and attention play a role. Further, processing for the (error) prosaccade and correct antisaccade compete for behavioural expression, a competition often modelled as a race between two accumulating signals (Munoz and Everling 2004; Noorani and Carpenter 2013). When one of these signals reaches threshold, its particular behaviour is exhibited (either an error prosaccade or a correct antisaccade). It is possible to bias the race in favour of one or other of the competing processes, and one way to do this is to change the fixation conditions. It has been known for some time that error rates in the antisaccade task are influenced by whether the fixation target is removed early (the gap paradigm—error rates are increased) or remains present (the overlap paradigm—error rates are decreased) when the saccade target appears (Fischer and Weber 1997). Forbes and Klein (1996) reported correct antisaccade latencies of 312, 292 and 265 ms for overlap, synchronous and gap conditions, respectively (a slightly smaller modulation than was observed for prosaccades), and directional error rates of 1.6, 4.7 and 13.3%. Munoz et al. (1998) reported a gap-overlap latency difference of approximately 50 ms for both pro- and antisaccade tasks, and error rates for the gap antisaccade task of 16% compared to 10% in an overlap version.

We found little evidence that either gap or overlap conditions affected latency or error rate in the MDOR task. There did appear to be reduction in error rate in overlap conditions, but this did not reach statistical significance and there was no difference in latency. There was certainly no evidence of effects of the magnitude reported for antisaccades. It is possible that as events at fixation are both temporally and spatially remote from the target to which participants have to saccade, and to which they presumably attend, the presence or absence of the fixation target has little bearing on MDOR task performance (this might be particularly the case in the 1000 ms condition). However, the lack of clear gap or overlap effects is more likely to be reflective of the absence of two competing tasks in the MDOR paradigm, in contrast to the antisaccade task. This competition has been argued to be sufficient to account for antisaccade behaviour in the absence of an explicit additional inhibitory signal (Cutsuridis 2017; Cutsuridis et al. 2007). Together, the absence of a large role for attention and working memory, and the absence of competing processes may be why we previously found no evidence of a correlation between antisaccade error rates and MDOR error rates (Wolohan and Knox 2014).

Top-down inhibitory control is, however, a prominent feature of the oculomotor system, with a number of cortical areas clearly able to exert an influence over the midbrain saccade control circuitry and saccade behaviour (Cieslik et al. 2016; Everling and Munoz 2000; Wegener et al. 2008). Coe and Munoz (2017) have recently suggested that both preparatory and reactive inhibition are involved in saccade control (as well as competitive inhibition). It has also been shown the inhibitory effects in the oculomotor network can be relatively long lasting and lead to effects of task context (Pierce and McDowell 2016; Weiler et al. 2014). In the absence of competitive inhibition, the MDOR task may provide a useful tool for probing these top-down inhibitory signals.

Powerful as such top-down inhibitory signals may be, clearly on some trials they are ineffective, and an error results. MDOR error rates are comparable with those observed in the antisaccade task. The 1000-ms display duration condition in the current experiments also provides an opportunity to probe the nature of errors (Fig. 5). An error response to target onset would be expected to have a latency consistent with a visually guided prosaccade, and would provide the clearest evidence of an inhibition failure. However, conceivably an error might be generated for some other reason. Successful inhibition of the target onset response could be followed by an anticipation of target offset. Evidence for this would be saccades initiated in the later part of the 1000-ms fixation period, perhaps just prior to target offset. We summarised performance in the 1000-ms condition in the synchronous MDOR task across participants using average frequency distribution histograms (see Knox et al. 2017 for a detailed description and discussion of the advantages of this approach). This analysis revealed that while errors occurred throughout the fixation period, there was a clear peak in the “low resolution” distribution (Fig. 5a; bin width 50 ms) 150 ms after target onset, consistent with reflexive, uninhibited responses to the target onset. This pattern of errors is consistent with what we observed previously (Wolohan and Knox 2014; see their Fig. 4). While the responses making up this peak comprised a relatively small proportion of responses overall, this proportion was markedly (and statistically significantly) higher than for two other epochs during the fixation period. While there was a small increase in errors prior to the target offset (during the “anticipatory” epoch), this was some way short of statistical significance.

When a narrower bin width was used for the error data (Fig. 5b; bin width 10 ms), the early peak was displaced earlier in time to 100 ms. It should be noted that because the average number of errors per bin per participant is low and variable (hence the wider 95% CI’s), with the narrower bin width there is little to statistically distinguish this early bin from the subsequent five bins (from 110 to 150 ms after target onset). While the higher resolution average distribution histogram for the correct responses suggested a degree of bimodality in the distribution (Fig. 5c; peaks at 250 and 290 ms), given the evident variability in these data, this needs to be treated with some caution.

Across three groups of different participants, we found no statistically significant difference in error rate or latency for the MDOR task. The levels of intersubject variability for these groups of healthy adults were broadly comparable with the antisaccade task. The intersubject variability for correct antisaccade latency reported in the literature, compared using the coefficient of variation (CoV: the standard deviation divided by the mean, expressed as a percentage), appears to vary from approximately 10–30%. Combining data for the synchronous MDOR task across our three datasets, and calculating a separate CoV for 200 and 1000 ms conditions, we calculated an overall CoV of 19 and 18%, respectively. Error rate is considerably more variable between different antisaccade experiments reported in the literature, and this is reflected in a much higher intersubject CoV, of the order of 65%. For the synchronous MDOR task, the CoV for error rate was 98 and 60% for 200- and 1000-ms conditions, respectively. Given this variability, establishing statistically robust differences in MDOR error rates between different groups will require relatively large numbers of participants. There would be value in investigating whether simple variations in stimulus conditions (e.g. the use of place holders for fixation and saccade target positions) reduce some of this variability.

The MDOR task is an apparently simple variant of a familiar saccade task that appears to provide a means of examining oculomotor inhibition with perhaps less involvement of processes such as attention and working memory than is the case for the antisaccade task. It remains to be seen whether in clinical populations known to have impaired inhibitory processing, MDOR performance is also impaired.
